# A long-term dataset of sable isotopes in rainfall at the North American monsoon region in southern Sonora, Mexico

**DOI:** 10.1016/j.dib.2022.108729

**Published:** 2022-11-07

**Authors:** Mayte F. Reyes-Hernández, Jesús A. Castro-López, Tonantzin Tarín, Jaime Garatuza-Payán, David H. Encinas-Yépiz, Enrico A. Yépez

**Affiliations:** a Departamento de Ciencias del Agua y Medio Ambiente, Instituto Tecnológico de Sonora, Ciudad Obregón, Sonora, México 85000; b Unidad Regional Guamúchil, Universisdad Autónoma de Occidente, Guamúchil, Sinaloa, México 81470; c Instituto de Ecología, Universidad Nacional Autónoma de México, México City, México 04510; d Sede Regional Sur de Sonora, Laboratorio Nacional de Geoquímica y Mineralogía, Instituto Tecnológico de Sonora, Ciudad Obregón, Sonora, México 85000

**Keywords:** Ecohydrology, Isotope hydrology, Oxygen 18, Deuterium, Local meteoric water line, Isoscapes

## Abstract

The objective of this work is to present a long-term dataset of water stable isotopes in rainfall samples from northwestern Mexico. These data is useful to generate a local meteoric water line as a reference tool for atmospheric and ecohydrological studies within the North American Monsoon region and to compare across the globe. This work shows the isotopic variation of the rainfall collected at a permanent location in Ciudad Obregon, Sonora, Mexico (27.511850, -109.956316), between 2014 and 2021. The isotopic composition of 138 rain samples was analyzed for both oxygen (δ^18^O) and deuterium (δ^2^H) with laser spectroscopy. The slope of the resulting local meteoric water line was m = 6.59 with an intercept of -1.15 (R² = 0. 91). During the monitored period at the studied region the presence of hurricanes, cold fronts and the hegemony of rainfall attributed to the North American Monsoon is recorded in the dataset.

**Specifications Table**

**Table:** 

Subject	Atmospheric sciences
Specific subject area	Isotope hydrology
Type of data	Table Figure
How the data were acquired	This is an original data set where rainwater samples were collected with a rain bucked prepared to avoid isotopic fractionation following the indication from the International Atomic Energy Agency (IAEA). We use a rainfall collector containing mineral oil, and samples from the collector were extracted within a maximum of 24 hours from the rain event and sometimes soon after precipitation ceased. Isotope analyses were carried with laser spectroscopy using working standards calibrated against the accepted Vienna Standard Meteoric Oceanic Water (VSMOW) for international reference and comparison.
Data format	Raw Analyzed Filtered
Description of data collection	We analyzed the isotopic composition of 138 rain samples collected at a permanent location with the aid of a rain bucket containing mineral oil. Samples were collected soon after every rainfall event between July 2014 and December 2021. Raw outputs from laser spectroscopes were confronted to International standards to adjust δ^2^H and δ^18^O values to the VSMOW scale and therefore all values presented at the repository are in δ notation as it is accepted by the international community.
Data source location	Rainfall was collected at one permanent location at a residential area in Ciudad Obregon, Sonora Mexico within the Cajeme municipality (27.511850, -109.956316) in the transition zone between the urban and agricultural area in the northeastern edge of the city.
Data accessibility	Repository name: ZENODO Data identification number: 10.5281/zenodo.7136588 Direct URL to data: https://doi.org/10.5281/zenodo.7136588

## Value of the Data

• This first long-term isotope monitoring dataset of rainfall in a semi-arid region of northwestern Mexico provides knowledge to understand the influence of North American monsoon on the region's water resources [1].
• Isotopic monitoring of rainfall is important to investigate surface atmospheric feedbacks, the recharge capacity of aquifers, and the availability of water for ecosystem function [1], [2], [3].
• A local meteoric water line in specific regions, such as areas limited by water, is useful to understand spatio-temporal variations of different ecohydrological processes as a function of climatic variability [4].
• Datasets containing multiple year data are useful to generate baseline information of meteorological phenomena which serves as a bench mark for validation of geostatistical models on which isoscapes are based [5].
• Isotope monitoring of rainfall water from northwestern Mexico can contribute to global databases such as the *Global Network of Isotopes in Precipitation (GNIP)* promoted by IAEA*.*

## 1 Objective

The fundamental goal for producing this dataset was to have a long-term record of the isotopic variation of rainfall at a very ecohydrological dynamic region within the area of influence of the North American monsoon in northwestern México. We believe that amounting original data to prior unexplored regions would advance synthesis work for better understanding of ecosystem and hydrological processes in highly seasonal regions.

## 2 Data Description

**Fig. 1:**
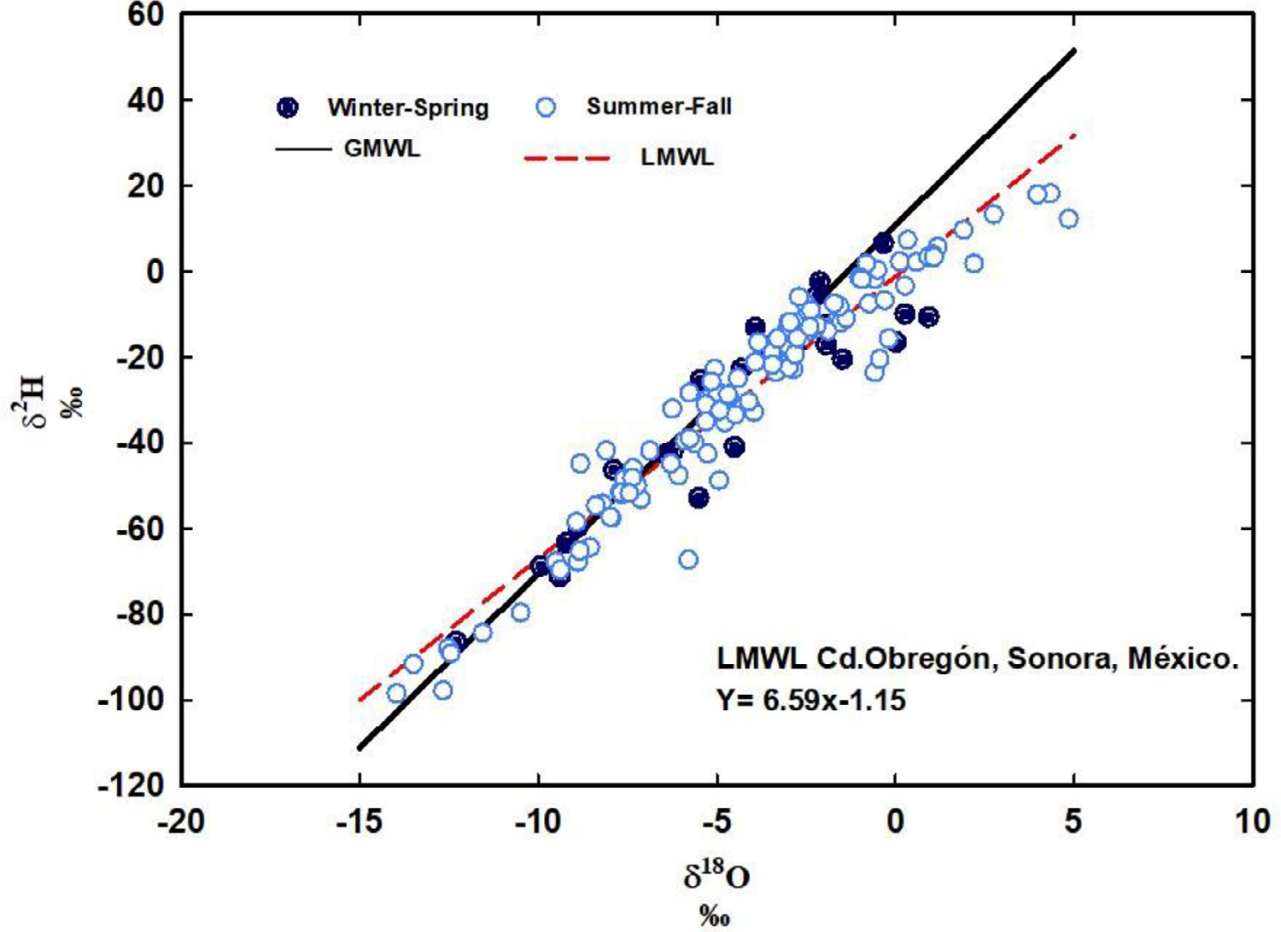
Local meteoric water line for Cd. Obregón, Sonora (dashed red), for the period from 2014 to 2021, the light blue symbols indicate the rain that occurs between June and November, the dark blue ones the rain that occurred between December and May.

We present a database containing a long-term record of stable isotope analyzes of rainfall water from the southern region of the state of Sonora, Mexico, which lies within the core region of the North American monsoon influence [6]. The information located in the repository [7] is an original dataset showing the oxygen and hydrogen isotopic concentration (δ^2^H and δ^18^O) following a standard confrontation of raw measurements against international accepted reference waters (i.e VSMOW). All data presented in the repository is in δ notation, that by convention with the stable isotope community is expressed in permil (‰) as the notation relies on:  *δ* = [(*R_sample_* /*R_standard_*) -1] × 1000, where *R_sample_* and *R_standard_* are molar ratios of heavy isotopes over light isotopes (^2^H/^1^H and ^18^O/^16^O) present in a sample and VSMOW, respectively. Of primary application of these data would be the production of a local meteoric water line (LMWL) for this highly seasonal and ecohydrological dynamic region [1,3]. In Fig. 1, were present the Local Meteoric Water Line for Ciudad Obregon, Sonora Mexico by simply relating the δ^2^H with δ^18^O from our dataset, a relation that is described by δ^2^H = 6.59* δ^18^O -1.15 ‰, (r^2^ =0.91) and for reference we indicate the so called Global Meteoric Water Line formalized by δ^2^H= 8.0* δ^18^O + 10 ‰ [8].

## 3 Experimental Design, Materials and Methods

A rainwater collection station was established in Ciudad Obregon, Sonora Mexico within the Cajeme municipality (27.511850, -109.956316), the station was located in a residential area, but in the transition zone between the urban and agricultural settings in the northeastern edge of the city. Rainwater samples were collected for each precipitation event that occurred between July 2014 and December 2021, for this purpose a collector with mineral oil was permanently deployed following the International Atomic Energy Agency (IAEA) recommendations to avoid evaporation [9]. Samples from the collector were extracted within a maximum of 24 hours from the rain event and sometimes soon after precipitation ceased and in all cases caution to avoid emulsification and evaporation was taken.

Isotope analysis were performed at the Ecohydrology and Stable Isotopes Laboratory of the Technological Institute of Sonora (ITSON) which is part of the National Laboratory of Geochemistry and Mineralogy (LANGEM; http://www.langem.org/). The Isotope analysis for the first two years (2014-2015) was carried on a DLT-100 Off-axis integrated cavity output spectroscopy (OA-ICOS) water isotope analyzer (Los Gatos Research, California, USA) and samples collected later (2016-2021) were analyzed using laser spectroscopy (Cavity Ring Down Spectroscopy (CRDS); (L2130-i, Picarro Inc., California, USA). The results obtained were normalized to international standards and calibrated with respect to VSMOW following the IAEA standard procedure. Isotope ratio values are expressed in δ notation in per mil (‰). The measurement uncertainty for the DLT-100 analyzes was ± 1.2 ‰ for δ^2^H and ± 0.4‰ for δ^18^O, and in the case of the L2130-i it was ± 0.6 ‰ for δ^2^H and ± 0.3 ‰ for δ^18^O.

## Ethics Statement

The present work did not involve human subjects, animals or information from social media platforms.

## CRediT authorship contribution statement

**Mayte F. Reyes-Hernández:** Methodology, Formal analysis, Investigation, Writing – original draft. **Jesús A. Castro-López:** Methodology, Formal analysis, Investigation, Writing – review & editing. **Tonantzin Tarín:** Methodology, Formal analysis, Investigation, Writing – original draft, Writing – review & editing. **Jaime Garatuza-Payán:** Methodology, Supervision, Writing – review & editing. **David H. Encinas-Yépiz:** Investigation, Writing – review & editing. **Enrico A. Yépez:** Conceptualization, Methodology, Supervision, Writing – review & editing.

## Declaration of Competing Interest

The authors declare that they have no known competing financial interests or personal relationships that could have appeared to influence the work reported in this paper.

## Data Availability

A long-term dataset of stable isotopes in rainfall at the North American monsoon region in southern Sonora, Mexico (Original data) (ZENODO).
